# Fish Oil Accelerates Diet-Induced Entrainment of the Mouse Peripheral Clock via GPR120

**DOI:** 10.1371/journal.pone.0132472

**Published:** 2015-07-10

**Authors:** Akiko Furutani, Yuko Ikeda, Misa Itokawa, Hiroki Nagahama, Teiji Ohtsu, Naoki Furutani, Mayo Kamagata, Zhi-Hong Yang, Akira Hirasawa, Yu Tahara, Shigenobu Shibata

**Affiliations:** 1 Laboratory of Physiology and Pharmacology, School of Advanced Science and Engineering, Waseda University, Wakamatsu-cho 2–2, Shinjuku-ku, Tokyo, Japan; 2 Central Research Laboratory, Nippon Suisan Kaisha Ltd., Nanakuni 1-32-3, Hachioji, Tokyo, Japan; 3 Department of Genomic Drug Discovery Sciences, Kyoto University, 46–29, Yoshida, Sakyo-ku, Kyoto, Japan; 4 Institute for Integrated Medical Sciences, Tokyo Women’s Medical University, Kawada-cho 8–1, Shinjuku-ku, Tokyo, Japan; Pennsylvania State University, UNITED STATES

## Abstract

The circadian peripheral clock is entrained by restricted feeding (RF) at a fixed time of day, and　insulin secretion regulates RF-induced entrainment of the peripheral clock in mice. Thus, carbohydrate-rich food may be ideal for facilitating RF-induced entrainment, although the role of dietary oils in insulin secretion and RF-induced entrainment has not been described. The soybean oil component of standard mouse chow was substituted with fish or soybean oil containing docosahexaenoic acid (DHA) and/or eicosapentaenoic acid (EPA). Tuna oil (high DHA/EPA), menhaden oil (standard), and DHA/EPA dissolved in soybean oil increased insulin secretion and facilitated RF-induced phase shifts of the liver clock as represented by the bioluminescence rhythms of PER2::LUCIFERASE knock-in mice. In this model, insulin depletion blocked the effect of tuna oil and fish oil had no effect on mice deficient for GPR120, a polyunsaturated fatty acid receptor. These results suggest food containing fish oil or DHA/EPA is ideal for adjusting the peripheral clock.

## Introduction

Circadian locomotor activity rhythm in rodents is entrained by an environmental light-dark cycle through activation of the suprachiasmatic nucleus (SCN), the primary oscillator, and by a fixed daily restricted feeding (RF) schedule through activation of a food-entrainment oscillator [1,2]. The daily increase of locomotor activity 2–3 h before RF is known as the anticipation activity rhythm [1,2]. Peripheral clock oscillators, located in peripheral organs such as the liver, are entrained via the RF paradigm [3,4]. Several mechanisms of RF-induced peripheral clock entrainment have been proposed and include increase in glucose levels [5], insulin release [6], adrenalin release [7], and thermogenesis [8]. Recently, Sato reported that insulin might be involved in feeding-induced mouse peripheral clock entrainment in vivo [9]. Insulin administration produced a time-dependent phase delay and advance of the circadian rhythm of clock gene expression in vitro [9].

Food components have also been identified as a factor in RF entrainment. We recently reported that highly digestible carbohydrates, which quickly increase blood glucose levels, have stronger entraining capacity than poorly digestible carbohydrates, and casein/amino acids may facilitate RF-induced entrainment [10,11]. The role of dietary oils in RF-induced entrainment of the liver clock has not been defined. Early experiments suggested mineral oil and plant oil injection could not mimic the increase in RF-induced anticipatory locomotor activity [12].

Many oils and lipids produce changes in fatty acid components. AIN-76 and AIN-93 are standard diets for rodents in nutrient experiments and contain soybean oil, comprised mainly of linoleic (50%) and oleic acid (20%). Many fatty acids are agonists of G-protein-coupled receptor (GPR)40 (FFAR1) [13,14] and GPR120 (FFAR4) [15]; linoleic and oleic acids exhibit low to moderate affinity for the GPR40 [13,14], while also showing high affinity to GPR120 [15]. In contrast, the primary fatty acid components of fish oil (such as tuna oil) are docosahexaenoic acid (DHA) (8–23%), eicosapentaenoic acid (EPA) (7–18%), palmitic acid (5–11%), and oleic acid (9–16%) (S1 Table). The agonistic activity of DHA/EPA for the receptor function of GPR40 and GPR120 was relatively higher than that of palmitic and oleic acid [13,14,15]. Unsaturated fatty acids such as omega-3 fatty acids DHA and EPA perform numerous biological functions including anti-inflammatory and anti-obesity/anti-diabetes activities [16]. DHA/EPA are agonists of GPR120 and GPR40 [14] in the lower ileum, upper colon, and pancreas, and cause insulin release through the production of glucagon-like peptide 1 (GLP-1) [14,17]. In addition, activation of GPR40 in the pancreas directly increases insulin secretion [13,18]. Furthermore, DHA/EPA facilitates the insulin signal cascade by activating IRS-1 and Akt kinase [19]. Therefore, substitution of soybean with fish oil may increase insulin levels through GPR40/GPR120 activation, and increased insulin may potentiate RF-induced entrainment of the liver clock.

These clock genes are the main components of molecular circadian oscillation and entrainment [20]. Therefore, we examined the ability of fish oil and a DHA/EPA-containing diet to increase insulin release, induce a liver clock phase-shift, and increase *Per2* expression in the liver. Finally, we defined the role of GPR120 in fish oil-induced phase shifts of the liver clock, insulin secretion, and acute induction of *Per2* gene expression in GPR120-deficient mice [21].

## Results

### RF-induced phase shifts of the liver clock by an AIN-93M diet containing fish oil or DHA and/or EPA dissolved in soybean oil

**Fig 1:**
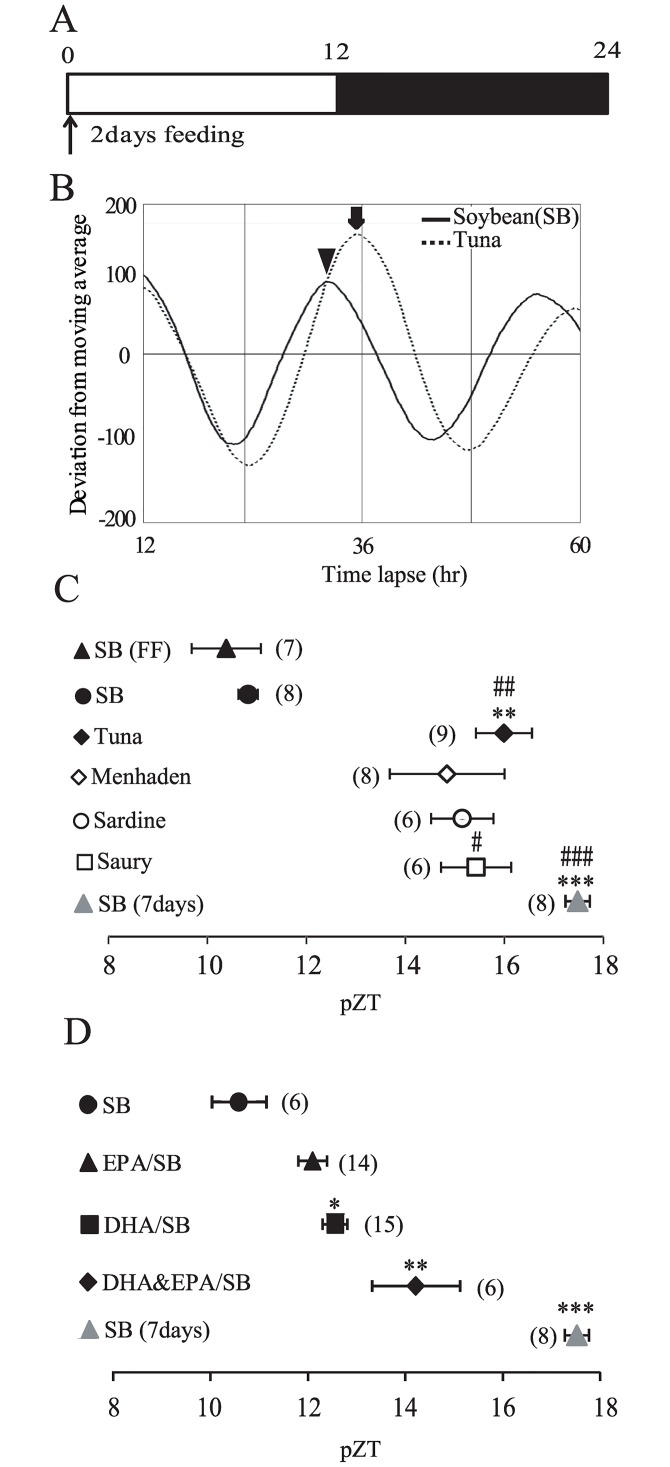
AIN-93M diet chow containing various fish oils or DHA/EPA dissolved in soybean oil and RF-induced phase shifts of the liver and SCN circadian clock. (A, C) mice were fed an AIN-93M diet tablet containing various substituted oils at ZT0 for 2 days or 7 days, and bioluminescence rhythm was recorded after sacrifice on Day 3 or Day 8. The horizontal axis indicates projected Zeitgeber time (pZT) at the peak of the bioluminescence rhythm. ZT0 is lights-on time and ZT12 is lights-off time in the housing room prior to sacrifice. (A) experimental protocol. RF was applied for 2 days at ZT0. (B) de-trended expression rhythms of the liver PER2::LUCIFERASE bioluminescence in mice under RF of an AIN-93M diet containing tuna oil (arrows) or soybean oil (arrow heads) for 2 days at ZT0. The horizontal line indicates time lapse. (C) magnitude of phase delay by fish oil-containing AIN-93M diet chow for 2 days at ZT0. A control experiment was prepared under free feeding conditions (closed triangle, FF). SB (7 days) shows the magnitude of the phase shifts by 7-day RF of soybean oil-containing AIM-93M at ZT0–ZT4 (Values are expressed as mean ± SEM. **P < 0.01, ***P < 0.001 (vs. SB, control chow, Dunn test). #*P* < 0.05, ###*P* < 0.001 [vs. SB, (FF), Dunn test]. (D) magnitude of phase-delay or phase-advance by DHA/EPA-containing AIN-93M diet chow. SB, soybean; FF, free-feeding. Numbers in parentheses indicate the number of tested mice. Values are expressed as mean ± SEM. *P < 0.05, **P < 0.01, ***P < 0.001 (vs. SB, control chow, Tukey-Kramer test).

Previously, we demonstrated liver clock phase-shift by altering the time of RF feeding; a large phase-shift was observed when RF was applied at Zeitgeber time (ZT) 0 [22]. ZT0 represents the end of feeding time under free-feeding conditions, when it is easy for the mouse to learn the feeding time. Fig 1A illustrates the RF experimental scheme. We examined whether a diet in which soybean oil is substituted with fish oil or DHA/EPA could potentiate RF-induced phase shifts of the liver clock. To avoid the celling effect of fish oil, food volume was reduced to 85–90% [10] After 24-h starvation, mice were fed 0.6 g/10 g bodyweight (BW) AIN-93M chow at ZT 0 on Day 1 and 0.75 g/10 g BW on Day 2; these mice showed no phase-delay in comparison to the free-feeding group (Fig 1B and 1C). Representative examples of the circadian rhythm of bioluminescence are shown in Fig 1B. RF consisting of various fish oil-containing diets at ZT0 for 2 days caused a significant liver clock phase delay (**P < 0.01, *** *P* < 0.001 vs. soybean oil, #*P* < 0.05 to ###*P* < 0.001 vs. FF) (Fig 1C). The magnitude of the RF-induced phase delay was larger in the tuna group than in groups fed diets containing other kinds of fish oils such as menhaden, sardine, and saury. As the phase delay induced by fish oil may be in response to DHA or EPA, an AIN-93M diet containing DHA and/or EPA was provided at ZT0 for 2 days. The levels of DHA, EPA, and DHA/EPA were adjusted in tuna oil, and DHA & EPA given at ZT0 caused a significant phase delay of the liver clock (*P < 0.05 to ***P < 0.001 vs. soybean oil group) (Fig 1D). The magnitude of the phase delay caused by DHA or EPA vs. non-DHA/EPA-containing soybean oil was 1.9 h and 1.4 h, respectively. The DHA/EPA combination caused a large phase delay (4.3 h) similar to that caused by tuna oil. The magnitude of the phase delay by tuna oil and DHA-containing soybean oil vs. soybean oil was 5.2 h (tuna oil) and 1.9 h (DHA), respectively. Two-day fish oil treatment yielded a phase delay comparable to that of a 7-day feeding of standard food from ZT0–ZT4 (Fig 1C and 1D). In summary, the diet containing fish oil or DHA/EPA produced a large effect on RF-induced phase delay in comparison to a diet containing soybean oil.

### RF-induced phase shift of the liver and SCN clock by the AIN-93M diet with tuna oil

**Fig 2:**
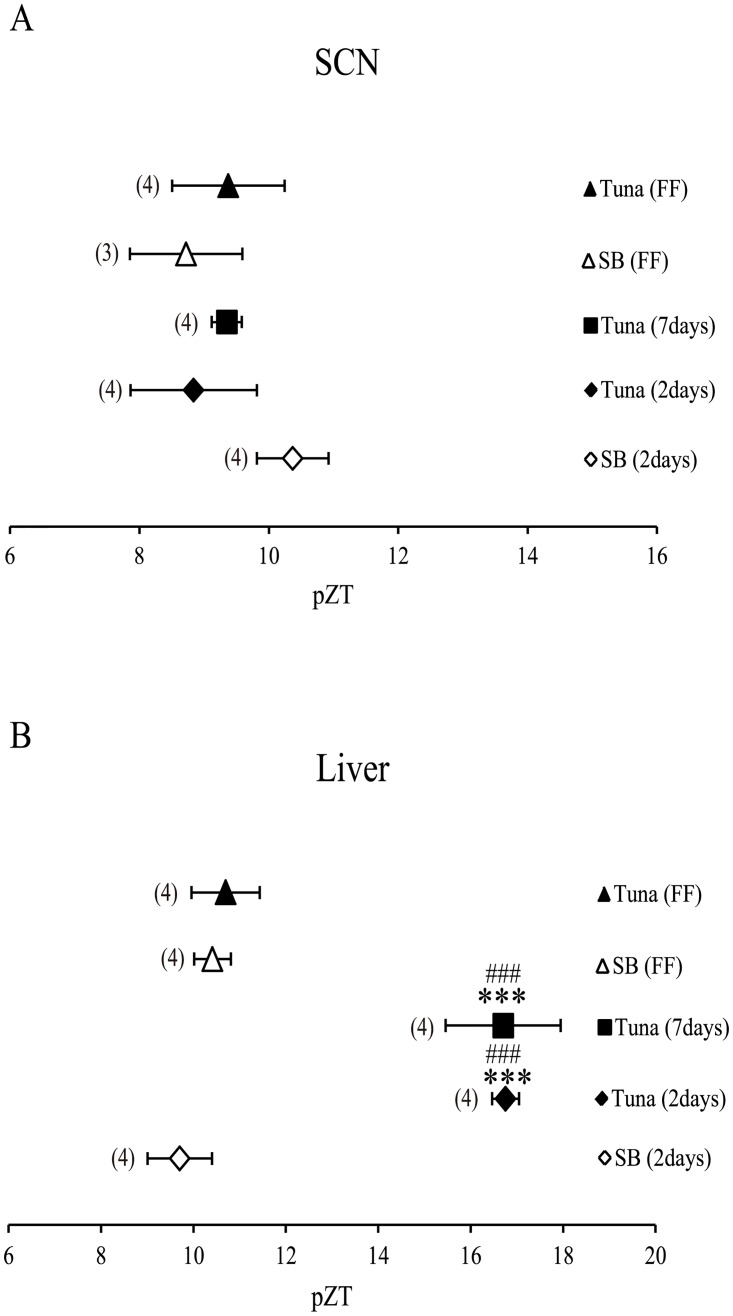
Effect of AIN-93M diet chow containing tuna oil on RF-induced phase shifts of the liver and SCN circadian clock. Mice were fed an AIN-93M diet containing tuna oil at ZT0 for 2 days or 7 days, and bioluminescence rhythm was recorded after sacrifice on Day 3 or Day 8. The horizontal axis indicates projected Zeitgeber time (pZT) at the peak of the bioluminescence rhythm. (A, B) simultaneous recording of bioluminescence from SCN (A) and liver (B). SB, soybean; FF, free-feeding. Numbers in parentheses indicate the number of tested mice. Values are expressed as mean ± SEM. ***P < 0.001 [vs. SB (2 days), control chow, Tukey-Kramer test]. ###*P* < 0.001 [vs. Tuna (FF) or SB (FF), Tukey-Kramer test].

Although a tuna oil-substituted diet caused a clear phase delay of the liver clock (Fig 1), we could not rule out the possibility that tuna oil and DHA/EPA also affected SCN, and that SCN conveys the output signals of phase delay to the peripheral clock. The liver clock was significantly phase-delayed by feeding a diet containing tuna oil for two or seven days (***P < 0.01 vs. soybean oil FF, ###P < 0.001 vs. tuna oil FF) (Fig 2B). The SCN clock was not significantly phase-delayed by 2-day or 7-day RF diet containing tuna oil. There were no significant differences in the phase delay of the SCN clocks in any group (F = 2.24, P = 0.12; one-way ANOVA), suggesting fish oil has no influence on the SCN clock.

### Substituted fish oil in the AIN-93M diet and insulin levels

**Fig 3:**
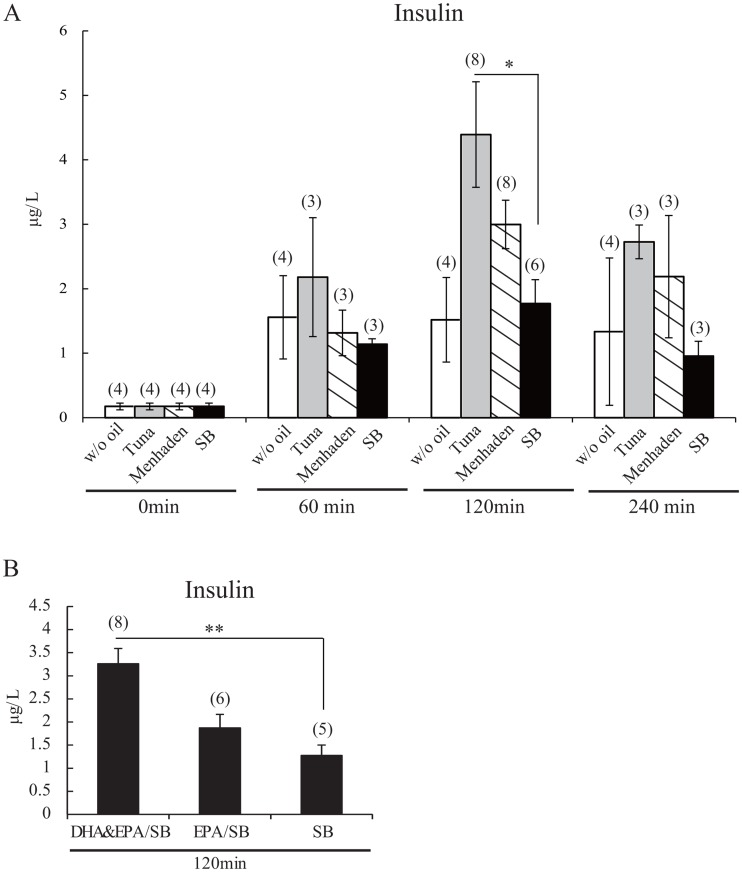
AIN-93M diet chow containing tuna oil and DHA/EPA and RF-induced insulin secretion. (A, B) on Day 2 under RF conditions, mice were fed an AIN-93M diet containing tuna, menhaden, soybean, DHA/EPA-containing soybean oil or without oil at ZT0; mice were sacrificed at 60 min, 120 min, or 240 min after feeding of fish oil (A) and 120 min after feeding of DHA/EPA (B). Serum insulin levels were measured. SB, soybean; w/o oil, without oil. Numbers in parentheses indicate the number of tested mice. Values are expressed as mean ± SEM. *P < 0.05 (vs. SB control chow, Tukey-Kramer test).

Insulin is an important hormone for RF-induced phase shifts of the liver clock [6,9], and omega-3 fatty acids increase insulin levels [14]. We examined whether the experimental feeding protocol similar to phase-delay experiment would increase serum insulin. Blood insulin levels were significantly and 2.5 times higher 120 min after feeding with tuna oil diets than feeding with soybean oil (*P* < 0.05) (Fig 3A). The DHA/EPA combination significantly increased insulin levels by 2.9-fold (*P* < 0.01) compared to the soybean oil group, but EPA-containing food alone caused an insignificant increase (Fig 3B), suggesting a parallel change in the magnitude of phase delay and increase in insulin levels.

### Fish oil and food-induced *Per2* mRNA expression in the liver

**Fig 4:**
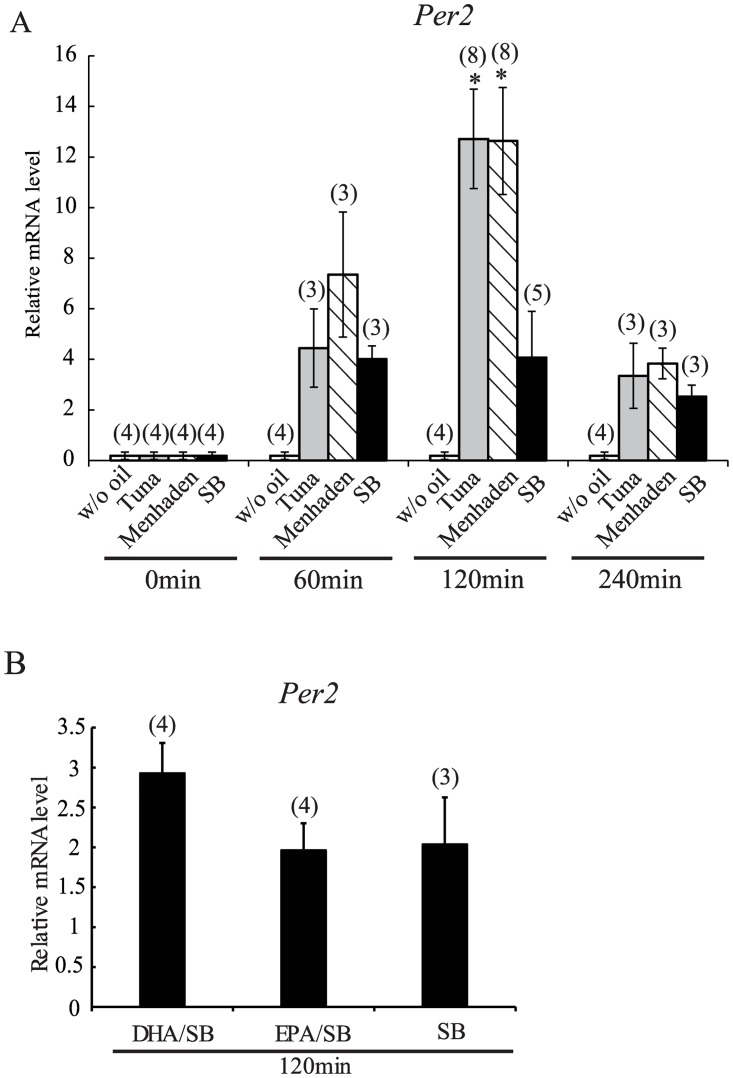
AIN-93M diet chow containing various fish oils and DHA/EPA and the RF-induced increase of *Per2* gene expression in the liver. (A, B) on Day 2 under RF conditions, mice were fed AIN-93M containing tuna, menhaden, soybean, DHA/EPA-containing soybean oil or without oil at ZT0; mice were sacrificed at 60 min, 120 min, and 240 min after feeding of fish oil (A) and 120 min after feeding of DHA/EPA (B). *Per2* gene expression was measured. The relative levels of expression were normalized to *GAPDH*. SB, soybean; w/o oil, without oil. Numbers in parentheses indicate the number of tested mice. Values are expressed as mean ± SEM. *P < 0.05 (vs. SB control chow, Tukey-Kramer test).

Re-feeding after starvation of mice fed *ad libitum* increases *Per2* gene expression in the liver [6,22,23]. To elucidate the mechanism mediating the phase of the liver *Per2* rhythm in response to fish oil vs. soybean oil feeding, we examined *Per2* gene expression in the liver 60, 120, and 240 min after re-feeding an AIN-93M diet containing tuna oil, menhaden oil, soybean oil, or DHA/EPA dissolved in soybean oil. *Per2* expression significantly increased by three-fold by 120 min after feeding the AIN-93M diet containing tuna oil (*P* < 0.05) (Fig 4A) compared to the soybean oil group (Fig 4B).

### Oral administration of fish oil or soybean oil containing DHA and/or EPA under free-feeding conditions slightly influences insulin level and phase shift

**Fig 5:**
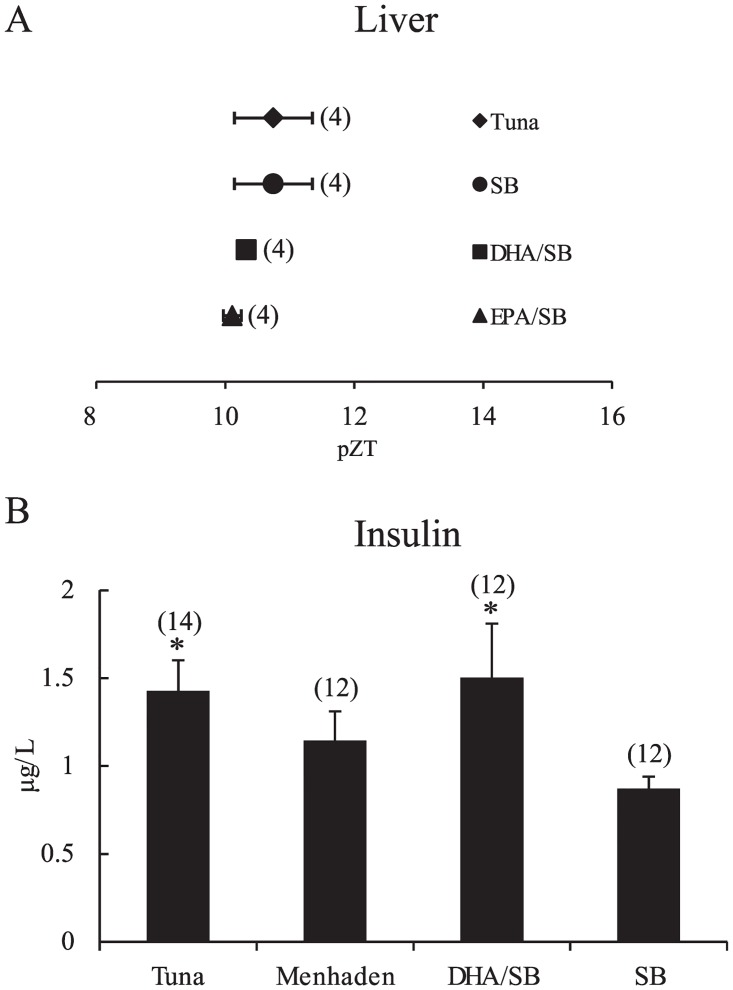
Effect of oral administration of fish oil or soybean oil containing DHA and/or EPA alone at daytime under free-feeding conditions on phase shifts and insulin level. (A) magnitude of phase shifts induced by tuna oil or soybean oil containing DHA and/or EPA administration at ZT0 for 2 days under free-feeding conditions. Tuna oil, soybean oil, DHA- or EPA-containing oil was administered at 0.034 ml/10 g BW for 2 days, and mice were sacrificed to examine the phase of bioluminescence of liver on Day 3. (B) tuna oil, menhaden oil, or DHA-containing oil was administered at 0.034 ml/10 g BW at ZT0 under free-feeding conditions, and mice were sacrificed 2 h after injection to examine serum insulin level. *P < 0.05 (vs. SB group, Tukey-Kramer test). Numbers in parentheses indicate the number of tested mice.

RF of a diet containing fish oil or DHA/EPA caused a large phase delay of the liver clock (Fig 1), but we do not know whether fish oil administration itself or in combination with the AIN-93M diet is necessary for this effect. When tuna oil- or DHA/EPA-containing soybean oil was orally administered to free-feeding mice at ZT0 for two days, no phase delay was observed (Fig 5A). Thus, the magnitude of phases in each group was similar to those of the free-feeding group. Although orally administrated tuna oil and DHA-containing soybean oil in free-feeding mice at ZT0 caused a significant increase in insulin 120 min after administration (Fig 5B), these values are small compared with combination of tuna oil and AIN-93M (Fig 3). Fish oil alone had a very weak effect on phase shift and increase in insulin levels.

### RF-induced phase shift of the liver clock by an AIN-93M diet containing fish oil in streptozotocin-injected insulin-depleted mice

**Fig 6:**
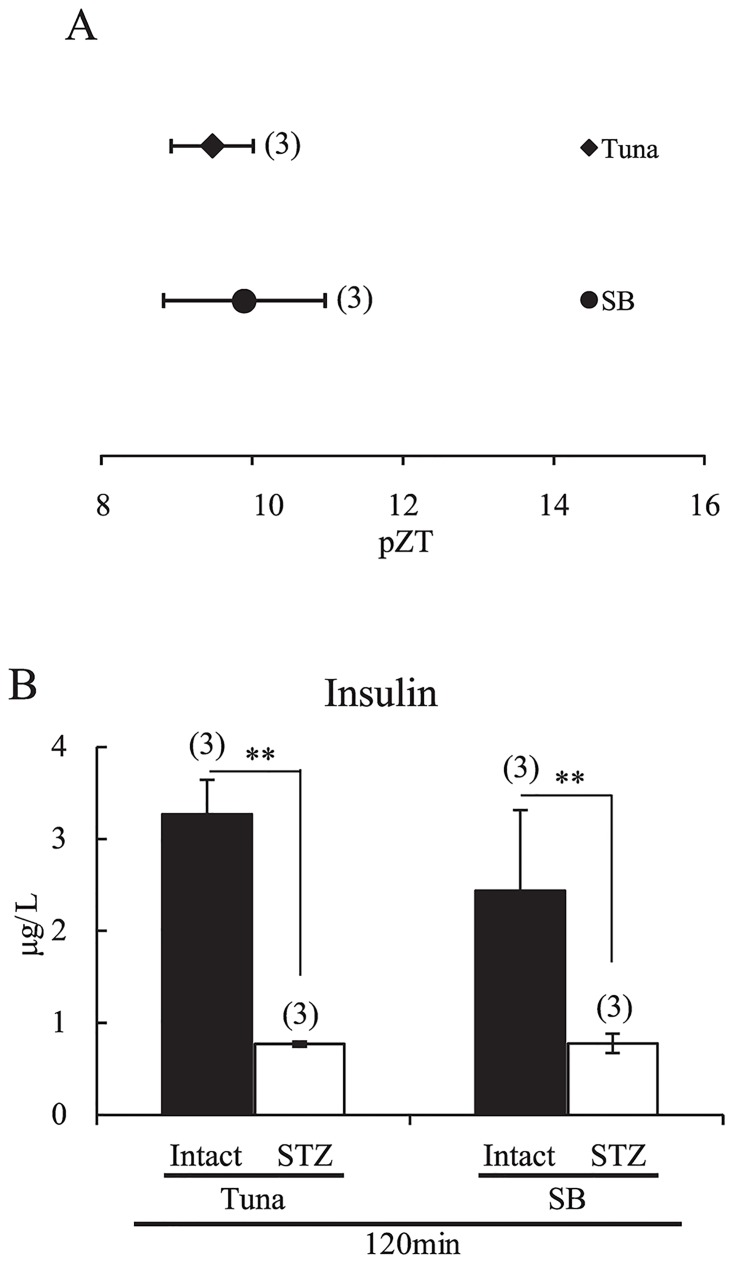
AIN-93M diet chow containing tuna oil and RF-induced phase-delay of the liver circadian clock and serum insulin in streptozotocin-pretreated mice. (A) bioluminescence rhythm recorded in the liver of STZ-treated mice fed AIN-93M diet chow containing tuna or soybean oil at ZT0 for 2 days. The horizontal axis indicates pZT at the peak of the bioluminescence rhythm. (B) on Day 2 under RF conditions, STZ-treated mice were fed AIN-93M containing tuna or soybean oil at ZT0 and sacrificed 120 min after feeding. Serum insulin levels were measured. Numbers in parentheses indicate the number of tested mice. Values are expressed as mean ± SEM. **P < 0.01 (vs. STZ-treated group, Tukey-Kramer test).

To understand the involvement of the insulin cascade in tuna oil-induced augmentation of phase-delay, insulin-depleted mice were prepared by pre-treatment with streptozotocin (STZ). In STZ-treated mice, the liver clock phase was not changed by feeding of tuna oil- or soybean oil-containing diet for two days at ZT0 (Fig 6A). STZ pre-treatment failed to increase insulin after feeding with the AIN-93M diet containing tuna oil or soybean oil (Fig 6B) vs. STZ-untreated intact mice. Thus, insulin release is necessary for fish oil-containing food-induced phase delay of the liver clock.

### Effect of fish oil on the sensitivity of insulin response using non-insulin-producing mouse embryonic fibroblasts cells

Insulin causes phase shifts of the circadian rhythm in hepatocytes and mouse embryonic fibroblasts (MEFs) in vitro [6,24]. Therefore, we asked whether fish oil affects insulin-induced phase delay of circadian rhythm in MEFs. Insulin administration caused phase delay compared with vehicle administration (S1A and S1B Fig) (time lapse difference between (a) and (b); S1C Fig). Tuna oil alone did not cause phase shifts in the circadian rhythms of MEFs (S1A and S1C Fig), nor did it change the magnitude of the phase delay induced by insulin application (S1B and S1C Fig).

### Restricted feeding-induced phase delay of the liver clock, insulin secretion, and clock gene expression by an AIN-93M diet containing fish oil in GPR120-deficient mice

We examined the role of GPR120 in fish oil-containing food-induced phase delay of the liver clock, expression of *Per2* gene, and insulin secretion in GPR120-deficient mice. Body weight and feeding volume were similar in wild-type and GPR120-deficient mice. As reported previously [21], daily activity patterns and activity counts were almost same in mice given normal diet and mice given diet containing fish oil (S2 Fig and S3 Fig).

**Fig 7:**
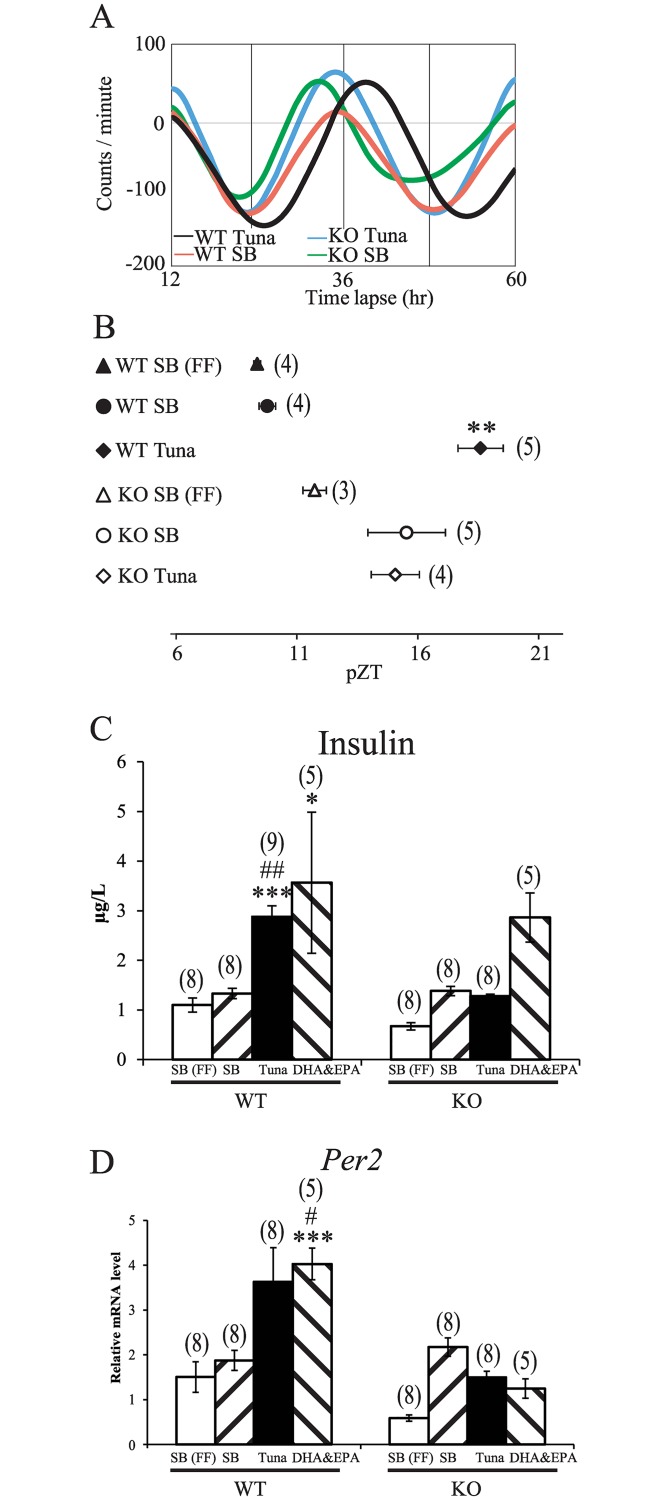
Phase shift of liver clock, insulin secretion, and clock gene expression in GPR120-deficient mice with tuna oil-containing diet. GPR120-deficient (KO) and wild-type (WT) mice were fed an AIN-93M diet tablet containing tuna oil at ZT0 for 2 days and bioluminescence rhythm was recorded after sacrifice on Day 3. The horizontal axis indicates projected Zeitgeber time (pZT) at the peak of the bioluminescence rhythm. ZT0 is lights-on time and ZT12 is lights-off time in the housing room prior to sacrifice. (A) de-trended data of expression rhythms of liver *PER2*::LUCIFERASE bioluminescence in mice under restricted-feeding (RF) of AIN-93M diet containing tuna or soybean oil. The horizontal line indicates time lapse. (B) magnitude of phase-delay by tuna or soybean oil in WT or KO mice. Values are expressed as mean ± SEM. **P < 0.01, (vs. WT SB group, Tukey-Kramer test). (C, D) on Day 2 under RF conditions, mice were fed AIN-93M containing tuna or soybean oil at ZT0, and sacrificed at 120 min to measure insulin and clock gene expression in the liver after feeding. (C) serum insulin levels. Values are expressed as mean ± SEM. *P < 0.05 *** *P* < 0.001 vs. WT SB group, ## *P* < 0.01 vs. WT SB (FF) group, Dunn test. (D) *Per2* gene expression. The relative levels of expression were normalized to *GAPDH*. *** *P* < 0.001 (vs. WT SB group), # *P* < 0.05 [vs. WT SB (FF) group Dunn test]. SB, soybean; FF, free feeding. Numbers in parentheses indicate the number of tested mice.

The phase of the liver clock under normal diet, free-feeding conditions was similar between wild-type and GPR120-deficient mice (Fig 7A and 7B). Tuna oil diet-induced phase delay was significant in wild-type mice, but attenuated in GPR120-deficient mice (Fig 7B). However, there was no significant difference in the magnitude of the phase shift by tuna oil between wild-type mice and GPR120-deficient mice. Daily activity patterns and the magnitude of anticipatory activity (activity increases 2–3 h before daily RF with standard or fish oil diet at ZT6-10 for 7 days) were similar in wild-type and GPR120-deficient mice (S2C, S2D and S2F Fig, S3C Fig, S3D and S3F Fig). In addition, target phase of the liver clock by daily RF with normal diet or diet containing fish oil at ZT6-10 for 7 days was similar (ZT6-ZT7) in wild-type and GPR120-deficient mice (S2G and S3G Fig).

Insulin secretion increased significantly by 120 min after feeding with tuna oil- or DHA/EPA-containing diet in wild-type, but not GPR120-deficient mice (Fig 7C). Acute induction of *Per2* expression by re-feeding of tuna oil- or DHA/EPA-containing diet was observed in wild-type mice, but attenuated in GPR120-deficient mice (Fig 7D).

## Discussion

Fish oil or DHA/EPA-containing chow enhanced RF-induced phase delay of the liver clock, increased insulin release, and potentiated *Per2* gene expression in the mouse liver. The involvement of insulin release in RF-induced phase shift was recently reported by Sato et al. [9]. Previous studies demonstrated that injecting mice with 100 nmol/g alpha-linolenic acid through a stomach tube after 24 h food deprivation increased insulin levels in the portal vein and inferior vena cava 30 min after injection [14]. DHA increases plasma insulin levels 180–240 min after intra-colonic injection [17]. ALA, DHA, and EPA administration in clonal pancreatic BRIN-BD11 cells produced dose-dependent increases in insulin secretion [25]. Intra-gastric administration of tuna oil and soybean oil containing DHA/EPA without diet chow caused a small increase in insulin, lower than that obtained after intake of AIN-93M containing fish oil, and failed to cause phase-delay of the liver clock (Fig 5). Therefore, DHA/EPA and fish oil potentiated the glucose-induced increase of insulin release and may be important for the potentiation of phase delay. The importance of insulin for phase entrainment of the liver clock and cellular clock has been reported [6,9,24]. Re-feeding after starvation increases *Per2* expression [6,22,23], and insulin injection increases *Per2* expression in the liver [6,23]. In this study, fish oil and DHA/EPA clearly potentiated diet chow-induced increases of *Per2* gene expression and may be the driving force behind RF-induced entrainment of the liver clock. In a recent paper, we demonstrated that highly digestible (vs. poorly digestible) starch mediated RF-induced entrainment of the liver clock; increasing blood glucose is key to this effect [10,26]. In the same study, oral injection of soybean oil did not induce entrainment [10]. Indeed, oral injection of glucose, but not vegetable oil or mineral oil, mimics the RF-induced anticipatory activity rhythm [12]. Therefore, we suggest that insulin-releasing foods such as sugar, digestible carbohydrates, and fish oil may participate in phase-shift of the peripheral clock through *Per2* gene expression.

Although the magnitude of the phase delay was greater in tuna (DHA/EPA: 23%/7%) than in the sardine (DHA/EPA: 13.5%/18.2%), saury (DHA/EPA: 14.5%/6.7%), or menhaden (DHA/EPA: 9.8%/12%), we could not conclude that the ratio of DHA/EPA was an important determinant of RF-induced phase delay of the liver clock. The DHA/EPA fatty acid components of fish oil constitute 20–30% of the total, depending on species (S1 Table). Total DHA/EPA content may be a pharmacological mediator RF-induced entrainment. Other functions of fish oil, such as their anti-inflammatory, anti-obesity, and cardio-protective effects, are also thought to be derived from DHA/EPA [27,28,29]. We added DHA and/or EPA to the soybean oil component of AIN-93M after adjusting the concentration to that found in tuna oil. Therefore, we could not make a direct comparison between DHA and EPA, although previous papers have reported DHA and EPA have different roles in various functions such as lipid metabolism and cardiovascular protection [30,31,32]. Furthermore, we may consider the ratio of n-3/n-6 PUFA in fish oil for RF-induced entrainment of the liver clock in future experiments.

Several studies have demonstrated an increase in GLP-1 secretion after administration of DHA and omega-3 fatty acids [14,17], and GLP-1 induced insulin secretion [25]. Therefore, GLP-1 may be involved in the fish oil containing food-induced potentiation of phase delay of liver clock. The role of GLP-1 in the regulation of circadian rhythm was examined by using exendin-4, a GLP-1 receptor agonist [33]. Injection of exendin-4 did not increase *Per2* gene expression like RF or insulin injection, but inhibited *Per1* gene expression; the authors concluded that exendin-4 modulates peripheral clocks via multiple mechanisms that are independent of refeeding. Although GLP-1 may be released after administration of fish oil or DHA/EPA, GLP-1 may not be involved in RF-induced entrainment of the liver clock.

The main fatty acid components of soybean oil are linoleic (50%), oleic (20%), and palmitic acid (10%); linoleic and oleic acids have relatively low to moderate affinity for GPR40 and palmitic acid has moderate affinity for GPR40 [14,15] (S1 Table). These fatty acids also have moderate affinity for the GPR120 receptor [14]. The main fatty acid components of tuna oil are DHA (23%), EPA (7%), palmitic acid (17%), oleic acid (21%), and palmitoleic acid (5%). The agonist activity of DHA/EPA for GPR40 and GPR120 was relatively high [14,15] (S1 Table). GPR40 and GPR120 play an important role in insulin release from the pancreas [13,25]. Oleic and palmitic acids are common to soybean and fish oil; therefore, the main differences are linoleic acid in soybean oil and DHA/EPA in fish oil. Thus, the DHA/EPA in fish oil may augment RF-induced entrainment of the liver clock. However, further experiments are necessary to define the importance of DHA/EPA compared to other omega-3 unsaturated fatty acids.

Our experiments in GPR120-deficient mice strongly suggested that GPR120 rather than GPR40 mediates fish oil-induced modification of the circadian clock, because almost all responses were fully attenuated in GPR120-deicient mice (Fig 7). Consistent with previous reports [21], food intake, body weight, and circadian activity rhythms did not differ between GPR120-deficient and wild-type mice. In addition, the increased anticipatory activity and target phase of the liver clock by RF with a standard diet or one containing menhaden oil were similar between wild-type and GPR120-deficient mice. These data suggest signaling of DHA/EPA-GPR120 is sufficient but not necessary for RF-induced phase shift and that dietary intake of fish oil may facilitate RF-induced phase shifting of the peripheral clock.

Agonists of GPR40 or GPR120 induce the release of cholecystokinin [34,35], and the role of cholecystokinin in the circadian rhythm has been reported [36]. Therefore, fish oil and DHA/EPA are possible mediators of the circadian rhythm through activation of cholecystokinin release. Thus, several pathways may mediate fish oil- and DHA/EPA-induced phase shifts in the peripheral clock.

Several studies have suggested that DHA/EPA are transported to the central nervous system [37] and improve neurological diseases [38,39]. Therefore, we examined whether RF-induced entrainment of the liver clock by fish oil occurs through the SCN clock. Tuna oil caused a significant phase delay of liver rhythm, but had a very small effect on SCN phase. Thus, fish oil-induced phase shifts of the liver clock may be independent of the SCN clock. The speed of re-entrainment of peripheral clock by phase-shift of light-dark schedule (jet-lag model) [40] may be facilitated by simultaneous phase-shift of the feeding schedule with food containing fish oil.

In summary, we discovered a new function of fish oil and DHA/EPA: they facilitate RF-induced entrainment of the peripheral clock through insulin secretion and activation of GPR120. From a practical perspective, meals supplemented with fish oil and/or DHA/EPA may help entrain the peripheral clock.

## Materials and Methods

### Animals and housing

PER2::LUCIFERASE knock-in mice [41] were bred in-house from PER2::LUCIFERASE homozygous male C57BL/6J mice and C57BL/6J female mice. From this crossing, we obtained PER2::LUCIFERASE heterozygous male mice weighing 25–30 g at the start of the experiment [10]. GPR120-deficient mice on a mixed C57Bl/6/129 background were generated by homologous recombination [21]. Some of these mice were mated with homozygous PER2::LUCIFERASE mice to obtain heterozygous PER2::LUCIFERASE GPR120-deficient mice. Insulin-deficient mice were prepared by injection with streptozotocin (STZ; 200 mg/kg, Sigma-Aldrich) and control mice were injected with saline. Animals with blood glucose levels >300 mg/dL after 12-h fasting were used in the experiment. The animal room had a controlled temperature of 22 ± 2°C, humidity 60% ± 5%, and a 12-h light/12-h dark cycle (lights on 08:00–20:00). ZT0 and ZT12 were used as lights-on and lights-off times, and pZT0 and pZT12 for *ex vivo* experiments, respectively. Light intensity at the surface of the cages was approximately 100 lux. Prior to the RF experiment, mice were fed normal, commercial rodent chow (Catalog #MF; Oriental Yeast Co., Tokyo, Japan) and provided with water *ad libitum*.

### Ethics statement

All experimental protocols were approved by the Committee for Animal Experimentation of the School of Science and Engineering at Waseda University (permission #09A11) and in accordance with the laws of the Japanese government.

### Recording of bioluminescence rhythm

Following the RF schedule, PER2::LUCIFERASE mice were sacrificed to record bioluminescence rhythmicity in the liver and SCN. Details of the ex vivo experiment were published previously [10]. Liver pieces and SCN slices were explanted in a 35-mm Petri dish with 1.3 mL DMEM. The cultures were incubated at 37°C and bioluminescence was monitored at 10-min intervals for 1 min using a dish-type luminometer (LumiCycle; Actimetrics, Wilmette, IL). First, the original data (1-min bins) were smoothed by an adjusting-averaging method with 2-h running means as described [10, 22, 26]. Then, the data set was de-trended by subtracting the 24-h running average from the raw data using R software. The highest peaks were identified on the waveform (Fig 1B).

### Mouse embryonic fibroblasts and insulin stimulation

The *in vitro* insulin stimulation experiment was performed using PER2::LUCIFERASE mouse MEFs [6]. MEFs were cultured in DMEM with vehicle (DMSO, 0.05%), fish/soybean oil dissolved in DMSO, insulin (100 nM, Sigma-Aldrich), or oil + insulin for 30 min, 40 h after dexamethasone stimulation (200 nM; Sigma-Aldrich). Drug treatment was performed for 30 min. Bioluminescence was measured for 2–3 cycles. Phase and amplitude of the last peak before, and the first peak after treatment were analyzed.

### Total RNA isolation and real time RT-PCR

Tissue mRNA was measured by real-time RT-PCR as described [6,22]. Mice were deeply anesthetized with ether and the liver was rapidly isolated. Total RNA (50 ng) was reverse-transcribed and amplified using the One-Step SYBR RT-PCR kit (Takara, Otsu, Japan) in a Step One Plus (Life Technologies Japan, Tokyo, Japan). Specific primer pairs were designed based on published data for *Gapdh Per2*. The *Gapdh*, *Per2* and were designed to cross exon-intron boundaries. Sequence of these genes were as follows: *Gapdh*, (forward) 5′-TGGTGAAGGTCGGTGTGAAC-3′ and (reverse) 5′-AATGAAGGGGTCGTTGATGG-3′, and *Per2*, (forward) 5′-TGTGTGCTTACACGGGTGTCCTA-3′ and (reverse) 5′-ACGTTTGGTTTGCGCATGAA-3′. Amplification product levels were normalized to *Gapdh*. Data were analyzed by the delta-delta Ct method and melt curve analysis indicated no amplification of non-specific products.

### Fish oils

Fish oils such as tuna, sardine, Alaska pollock, and pacific saury were obtained from Nippon Suisan Kaisha (Tokyo, Japan), and menhaden, soybean, and coconut oil were purchased from Sigma-Aldrich. DHA ethyl ester and EPA ethyl ester were obtained from Chemport (Taejon, Korea). CPFA-D85E02 EE (85% DHA/2% EPA) was used as the source of DHA, and CPFA-E92D00 EE (92% EPA/0% DHA) was the source of EPA. DHA and EPA contents were adjusted to those of tuna oil: DHA (23%) and/or EPA (7%). All oil and DHA/EPA was stored at −80°C after air purge by N_2_ gas.

### Determination of fatty acid components

The fatty acid composition (S1 Table) of each dietary oil was determined after methylation with 14% boron trifluoride-methanol solution (BF_3_-methanol, Sigma-Aldrich) at 80°C for 30 min. Fatty acid methyl esters were quantified by gas chromatography using an Agilent 6890N Network Gas Chromatograph System (Agilent Technologies Japan, Ltd., Tokyo, Japan) equipped with a split injector, an FID detector, and a fused silica capillary column (30 m × 0.25 mm I.D. × 0.25 μm film thickness, J & W Scientific, Agilent Technologies). The column temperature was raised from 180°C to 230°C at 3°C/min, and the injector and detector temperature was set at 250°C. Data were collected with a GC Chemstation (Agilent Technologies). Methyl esters were identified by comparing the retention times of standard fatty acid methyl esters (Nu-Chek Prep, Elysian, MN) as described [42].

### Preparation of food tablets for restricted feeding

For the RF experiments, we prepared diet tablets using a tableting machine (HANDTAB-100; Ichihashi-seiki, Kyoto, Japan). For the control, AIN-93M formula diet was prepared (Oriental Yeast Co. Ltd., Tokyo, Japan; composition: 14% casein, 0.3% L-cysteine, 47% corn starch, 15% gelatinized corn starch, 10% sucrose, 4% soybean oil, 5% cellulose powder, 3.5% AIN-93 mineral mixture, 1% AIN-93 vitamin mixture, 0.25% choline bitartrate, and 0.0008% tert-butyl hydroquinone). For substitution experiments, the oil component (soybean oil) of the diet was substituted with various fish oils, soybean oil, and soybean oil containing DHA and/or EPA.

### Experimental procedure for phase shift of bioluminescence rhythm, insulin, and clock gene expression

To allow the mice to adapt to the control diet, their diet was changed 3–4 days prior to the experiment. After 24 h food deprivation, mice were applied to RF paradigms such as 0.6 g/10 g BW on the first day and 0.75 g/10 g BW on the second day at ZT0 similar to our previous report [10]. However, in this experiment, the food volume was 85–90% of that used in our previous protocol [10], because a fish oil-containing diet may facilitate phase-shift and lead to a ceiling effect. Two hours after the food tablet was provided, we checked for consumption. Many mice ate the entire tablet within 120 min, but those that did not were excluded from the bioluminescence rhythm experiment.

On Day 2 under RF conditions, food tablets were given to the mice; 60, 120, or 240 min after presentation each mouse was sacrificed for insulin measurement. To examine the effect of fish oil in the absence of food, fish or soybean oil (0.034 mL/10 g BW, 4% suitable volume of diet chow) was orally administered at ZT0 under free-feeding conditions.

### Recording of locomotor activity and RF-induced anticipatory activity and entrainment of the liver clock

PER2::LUCIFERASE knock-in wild-type or GPR120-deficient mice were housed individually during measurement of locomotor activity. General locomotor activity was recorded with an infrared radiation sensor (F5B, Omron, Tokyo, Japan) and analyzed with CLOCKLAB software (Actimetrics, Wilmette, IL). Percent changes in activity before and after RF were calculated as 100 × (1-h bin activity/daily total 24-h activity). The magnitude of anticipatory activity was evaluated by comparing the mean percent activity for ZT3-ZT6 7 days after and before the RF schedule. On the eighth day after RF, the mice were sacrificed at ZT3 to record bioluminescence in *ex vivo* liver tissue.

### Statistical analysis

All data are expressed as means + or ± SEM (standard error of the mean). Statistical analysis was performed using GraphPad Prism version 6.03 (GraphPad software, San Diego, CA, USA). We determined whether the data showed a normal or non-normal distribution and equal or biased variation assessed by the D’Agostino-Pearson/Kolmogorov-Smirnov and F value tests, respectively. Parametric analysis was conducted by one-way analysis of variance ANOVA with Tukey-Kramer test or Student t-test for post hoc analysis, and non-parametric analysis was performed using the Kruskal-Wallis/Friedman test with a Dunn’s test for post hoc analysis.

## Supporting Information

S1 Fig Effect of fish oil on insulin-induced phase delay of bioluminescence rhythm and the phase-shifting effect of fish oil on bioluminescence rhythm from MEFs of PER2::LUCIFERASE knock-in mouse. (A) tuna oil (blue line) and soybean oil (brown line), and DMSO (final concentration, 0.05%; green line) did not affect the phase of rhythm compared to DMSO (final concentration, 0.05%; green line). (B) insulin administration-caused phase delay compared to peak (a). Co-administration of insulin and tuna oil (blue line), soybean oil (brown line), and DMSO (final concentration, 0.05%; green line) did not affect the phase delay following insulin administration. (A,B) representative de-trended data of expression rhythms of bioluminescence in MEFs. Arrow heads indicate oil and/or insulin application. The horizontal line indicates the time lapse. Peak (a) and peak (b) indicate rhythm peak before and after oil application, respectively. (C) summarized data of peak (a) and peak (b). Vertical axis indicates the time lapse at peak (a) and peak (b) of the bioluminescence rhythm. Values are expressed as mean ± SEM. *P < 0.05 (vs. peak (a), Tukey-Kramer test). Numbers in parentheses indicate the number of tested dishes.
(EPS)

S2 Fig Effect of restricted feeding of standard diet during daytime on anticipatory activity rhythm and phase-advance of liver clock in GPR120 deficient mice. PER2::LUCIFERASE knock-in Wild-type (WT) or GPR120 deficient (KO) mice were prepared. (A, B) representative double plotted actgrams of locomotor activity in WT and KO mice, respectively. Mice were given normal standard food by free-feeding (FF, vertical dark line) for 7 days or restricted-feeding (RF, vertical red line) during ZT6-ZT10 for 7 days. Horizontal white and black bars exhibit environmental light-dark period. (C, D) percent change in activity (%) before (closed circle, corresponding period in vertical black line, in Fig A and B) and after RF (open circle, corresponding period in vertical red line, in Fig A and B) for 7 days. Values are expressed as mean ± SEM from 4 mice. (E) percent locomotor activity during light period (white column) and dark period (black column) in WT and KO mice. Values are expressed as mean ± SEM from 4 mice. **P < 0.01 (vs. dark period, Student t-test). (F) anticipatory activity counts. Vertical values, mean percentage activity during ZT3-ZT6 (Horizontal rectangular box, in Fig C and D) for 7 days under FF or RF conditions. Values are expressed as mean ± SEM from 4 mice. **P < 0.01 (vs.FF, Student t-test). (G) magnitude of phase-advance of liver bioluminescence rhythm by RF of standard AIN-93M diet for 7 days in WT and KO mice. The horizontal axis indicates projected Zeitgeber time (pZT) at the peak of the bioluminescence rhythm. ZT0 is lights-on time and ZT12 is lights-off time in the housing room prior to sacrifice of the mice.
(EPS)

S3 Fig Effect of restricted feeding of menhaden oil containing diet during daytime on anticipatory activity rhythm and phase-advance of liver clock in GPR120 deficient mice. PER2::LUCIFERASE knock-in Wild-type (WT) or GPR120 deficient (KO) mice were prepared. (A, B) representative double plotted actgrams of locomotor activity in WT and KO mice, respectively. Mice were given AIN-93M containing menhaden oil by free-feeding (FF, vertical dark line) for 7 days or restricted-feeding (RF, vertical red line) during ZT6-ZT10 for 7 days. Horizontal white and black bars exhibit environmental light-dark period. (C, D) percent change in activity (%) before (closed circle, corresponding period in vertical black line, in Fig A and B) and after RF (open circle, corresponding period in vertical red line, in Fig A and B) for 7 days. Values are expressed as mean ± SEM from 4 mice. (E) percent locomotor activity during light period (white column) and dark period (black column) in WT and KO mice. Values are expressed as mean ± SEM from 4 mice. **P < 0.01 (vs. dark period, Student t-test). (F) anticipatory activity counts. Vertical values, mean percentage activity during ZT3-ZT6 (Horizontal rectangular box, in Fig C and D) for 7 days under FF or RF conditions. Values are expressed as mean ± SEM from 4 mice. **P < 0.01 (vs.FF, Student t-test). (G) magnitude of phase-advance of liver bioluminescence rhythm by RF of AIN-93M diet containing menhaden oil for 7 days in WT and KO mice. The horizontal axis indicates projected Zeitgeber time (pZT) at the peak of the bioluminescence rhythm. ZT0 is lights-on time and ZT12 is lights-off time in the housing room prior to sacrifice of the mice.
(EPS)

S1 Table Fatty acid composition of dietary oils and pEC50 values of fatty acids tested in HEK 293 cells stably expressing GPR120 and GPR40. Values correspond to the mean of three separate samples processed independently. ΣSaturated: Total saturated fatty acid;ΣMUFA: Total monounsaturated fatty acids;ΣPUFA: Total polyunsaturated fatty acids; ND: Not detected; IA: Inactive, no response at 100μM. #1 Data from Ref.14, #2 Data from Ref.17.
(DOCX)
