# Supplementary material for: Fish Oil Accelerates Diet-Induced Entrainment of the Mouse Peripheral Clock via GPR120
Source: PLoS One. 2015 Jul 10;10(7):e0132472. doi: 10.1371/journal.pone.0132472 (PMC4498928; doi:10.1371/journal.pone.0132472)
Supplement: S1 Table — Values correspond to the mean of three separate samples processed independently. ΣSaturated: Total saturated fatty acid;ΣMUFA: Total monounsaturated fatty acids;ΣPUFA: Total polyunsaturated fatty acids; ND: Not detected; IA: Inactive, no response at 100μM. #1 Data from Ref.14, #2 Data from Ref.17. (DOCX) [file pone.0132472.s004.docx]

|  | |  |  |  |  |  |  |  |  |
| --- | --- | --- | --- | --- | --- | --- | --- | --- | --- |
| S1 Table Fatty acid composition of dietary oils and pEC50 values of fatty acids tested in HEK 293 cells stably expressing GPR120 and GPR40. | | | | | | | | |  |
| Fatty acids (%) | Plant oils | | Fish oils | | | | | pEC_50_ | |
|  | Coconuts | Soybean | Alaska pollack | Sardine | Saury | Tuna | Menhaden | GPR120 (#1) | GPR40(#2) |
| C10:0 | 5.8 | ND | ND | ND | 0.03 | ND | 0.02 | IA | 4.85 |
| C12:0 | 49.6 | 0.02 | 0.05 | 0.04 | 0.1 | 0.05 | 0.2 | IA | 4.92 |
| C14:0 | 20.3 | 0.07 | 4.4 | 5.8 | 3.9 | 3.2 | 7.5 | 4.53 | 4.84 |
| C16:0 | 10.5 | 9.8 | 10.4 | 13.7 | 9.1 | 17.3 | 23.4 | 4.28 | 5.3 |
| C18:0 | 3.2 | 3.9 | 2.6 | 2.4 | 2.1 | 4.2 | 3.9 | 4.74 | 4.78 |
| C20:0 | 0.1 | 0.3 | 0.1 | ND | 0.9 | 0.2 | 0.2 | IA | 4.21 |
| ∑Saturated | 89.5 | 14.1 | 17.6 | 21.9 | 16.1 | 25.0 | 35.2 |  |  |
| C16:1n-7 | 0.02 | 0.02 | 10.5 | 8.5 | 4.9 | 5.3 | 9 | 5.49 | 4.86 |
| C18:1 n-9 | 7.6 | 21.6 | 16 | 14.2 | 6.2 | 21.1 | 9.6 | 4.48 | 4.39 |
| C20:1 n-7 & n-9 | 0.05 | 0.2 | 11.4 | 3.4 | 20.6 | 3 | 1.5 | - | - |
| C22:1 n-11& n-9 | ND | ND | 13.6 | 3 | 22.7 | 1.9 | 0.04 | - | - |
| ∑MUFA | 7.7 | 21.8 | 51.5 | 29.1 | 54.4 | 31.3 | 20.1 |  | - |
| C18:2 n-6 | 2.2 | 52.7 | 1 | 1.2 | 1.5 | 1.2 | 1.4 | - | 5.02 |
| C20:2 n-6 | ND | 0.03 | 0.3 | ND | 0.2 | ND | 0.2 | - | 4.97 |
| C20:4 n-6 | ND | ND | 0.5 | 1.5 | 0.9 | 2 | 1.5 | - | 4.92 |
| ∑n-6 PUFA | 2.2 | 52.73 | 1.8 | 2.7 | 2.6 | 3.2 | 3.1 |  |  |
| C18:3 n-3 | 0.02 | 6.9 | 0.7 | 0.7 | ND | 0.5 | 1.5 | 6.37 | 4.9 |
| C20:4 n-3 | ND | ND | 0.7 | 0.9 | 1.2 | 10.6 | 1 | - | - |
| C20:5 n-3 | 0.03 | 0.4 | 12.3 | 18.2 | 6.7 | 7 | 12 | 5.55 | 5.17 |
| C22:5 n-3 | ND | ND | 1.1 | 2.6 | 2.7 | 1.5 | 0.07 | 4.58 | 5.33 |
| C22:6 n-3 | ND | ND | 7.9 | 13.5 | 14.5 | 23.3 | 9.8 | 5.41 | 5.37 |
| ∑n-3 PUFA | 0.1 | 7.3 | 22.7 | 35.9 | 23.9 | 42.9 | 24.4 |  |  |
| Values correspond to the mean of three separate samples processed independently. | | | | | |  |  |  |  |
| ∑Saturated: Total saturated fatty acid;∑MUFA: Total monounsaturated fatty acids;∑PUFA: Total polyunsaturated fatty acids; | | | | | | | | |  |
| ND: Not detected; IA: Inactive, no response at 100μM. | | | |  |  |  |  |  |  |
| #1 Data from Ref.14, #2 Data from Ref.17 | | |  |  |  |  |  |  |  |
